# The Contribution of Lysosomes to DNA Replication

**DOI:** 10.3390/cells10051068

**Published:** 2021-04-30

**Authors:** Joanna Maria Merchut-Maya, Apolinar Maya-Mendoza

**Affiliations:** 1 DNA Replication and Cancer Group, Danish Cancer Society Research Center, DK-2100 Copenhagen, Denmark; jomema@cancer.dk; 2 Genome Integrity, Danish Cancer Society Research Center, DK-2100 Copenhagen, Denmark

**Keywords:** lysosomes, autophagy, mTORC1, DNA synthesis, cancer

## Abstract

Lysosomes, acidic, membrane-bound organelles, are not only the core of the cellular recycling machinery, but they also serve as signaling hubs regulating various metabolic pathways. Lysosomes maintain energy homeostasis and provide pivotal substrates for anabolic processes, such as DNA replication. Every time the cell divides, its genome needs to be correctly duplicated; therefore, DNA replication requires rigorous regulation. Challenges that negatively affect DNA synthesis, such as nucleotide imbalance, result in replication stress with severe consequences for genome integrity. The lysosomal complex mTORC1 is directly involved in the synthesis of purines and pyrimidines to support DNA replication. Numerous drugs have been shown to target lysosomal function, opening an attractive avenue for new treatment strategies against various pathologies, including cancer. In this review, we focus on the interplay between lysosomal function and DNA replication through nucleic acid degradation and nucleotide biosynthesis and how these could be exploited for therapeutic purposes.

## 1. Introduction

Organismal and cellular homeostasis is achieved by the balance between anabolic and catabolic processes. Lysosomes are important for metabolic homeostasis maintenance in normal conditions, but they also provide a constant supply of metabolites in transformed cells [1]. Lysosomes are intracellular organelles that degrade cargos from the endocytic pathways and are required for the degradation of intracellular components tagged for recycling [2]. Lysosomes contain about 50 hydrolases, including nucleases, peptidases, lipases and glycosidases, that degrade several different molecules. The acidic luminal space of the lysosome, with a pH of around 4.5, is surrounded by a single phospholipid bilayer [3]. The acidic pH is maintained by proton-pumping ATPases. In the endoplasmic reticulum, precursors of the lysosomal enzymes are synthesized and modified in the Golgi apparatus [4]. In the trans-Golgi network, post-translationally modified enzymes bind to mannose-6-phosphate receptors and are taken into clathrin-coated vesicles. Later, these vesicles lose the clathrin coat and fuse with double-membrane structures, the autophagosomes. The process of delivering intracellular components for degradation into lysosomes is known as macroautophagy (commonly referred to as autophagy). Autophagy is often highly active in cancer, promoting cell survival during nutritional stress and recycling damaged cell components [5].

During starvation, autophagosomes accumulate and trap their cargo, which is then delivered to the lysosome via membrane fusion [6]. Enzymes located in the lumen of the lysosome degrade the cargo from the autophagosome and release the ensuing metabolites for further use. The contribution of autophagy to balance the anabolic and catabolic processes in the cell is compelling. Nevertheless, other endocytic pathways can be also relevant; for instance, lysosomes can fuse directly with mitochondrial-derived vesicles to remove only damaged proteins and lipids from the otherwise intact mitochondria [7].

Lysosomes play an important role in sensing the availability of nutrients by the cell, which is critical for cellular growth and proliferation. A better understanding of the mechanisms that regulate lysosomal function, the source and the type of cargo trafficked to the lysosomes could help to determinate differences in the use of nutrients between normal and transformed cells. These differences could be explored for therapeutic purposes, to tackle various pathologies, including metabolic disorders and cancer, with relevant inhibitors or dietary intervention.

Early studies of the lysosomal function led to the identification of lysosomotropic agents, drugs that are able to penetrate the lysosomes. The antimalarial drug, quinacrine, accumulates in the lysosomes in a pH-dependent manner [8]; thus, it has been used to test lysosomal permeability. The contribution of lysosome function to cell growth and DNA synthesis was suggested in the 1960s. Bastos and Nunes showed that DNA synthesis of cells growing in vitro is inhibited in the presence of quinacrine [9]. The effect of quinacrine on cell proliferation may be due to its DNA binding properties [10]. These early observations suggested that chemical components that target lysosomes may also target nucleic acid metabolism in the cell. In this review, we provide evidence of the potential role of lysosomes in regulating DNA synthesis through cell metabolism regulation. We also propose a notion that drugs affecting lysosomal activity could impact nuclear function.

## 2. mTORC1 Activity and DNA Synthesis

The mammalian target of rapamycin complex 1 (mTORC1) is a main integrator and effector of growth and nutrient signals in the cell. mTORC1 is a dimer with a weight of approximately 1 MDa. The complex consists of mTOR, a serine/threonine protein kinase related to the PI3K family [11], and several regulatory proteins. To form an active complex, mTORC1 needs to be localized to the cytosolic surface of the lysosome membrane, most likely in an amino acid dependent manner, where it interacts with the proteins Rag and Rheb. In the absence of amino acids, Rag proteins are in a conformation unable to bind to mTORC1, preventing activation of the complex. An optimal level of intracellular amino acids promotes the interaction of Rag proteins with mTORC1 [12]. Rag proteins bring mTORC1 to its activator Rheb, switching on the mTORC1 kinase activity [13,14]. Activated mTORC1 inhibits autophagy through the phosphorylation of several autophagosome-initiation factors [15].

mTORC1 is regulated also by the glucose level. Glucose deprivation activates AMPK (adenosine monophosphate activated protein kinase), which inhibits mTORC1 through the phosphorylation of the regulator Raptor [16]. AMPK is an intracellular energy sensor activated by high ratios of AMP/ATP (adenosine monophosphate/adenosine triphosphate) and ADP/ATP (adenosine diphosphate/ATP). AMPK inhibits anabolic pathways and suppresses cell growth by inhibiting mTORC1 [17]. Furthermore, glucose deprivation can inhibit mTORC1 through the inhibition of Rag, independently of AMPK inactivation [18]. Interestingly, upon glucose starvation, a proportion of AMPK is translocated from the cytoplasm to the lysosomes. A model proposes that the recruitment of AXIN and LKB1 to the lysosome causes the dissociation of mTORC1 from the membrane surface. LKB1 then phosphorylates AMPK, triggering its activation. In response to energy stress, AMP can additionally enhance the mTORC1–AXIN interaction, which allows the activation of AMPK [19]. In general, it is considered that mTORC1 plays an opposing role to AMPK. mTORC1 stimulates anabolic pathways under high-nutrient conditions, while AMPK under low-nutrient conditions induces catabolic pathways. The role of AMPK in the regulation of metabolism involves inhibition of anabolism to minimize ATP consumption and stimulation of catabolism to facilitate ATP production [20].

It has been suggested that mTORC1 is involved directly in the synthesis of purines and pyrimidines to support DNA replication and transcription [21]. mTORC1 is inhibited upon depletion of purine nucleotides and is particularly sensitive to changes in adenylate nucleotide levels. Prolonged depletion of intracellular purines resulted in Rheb degradation [22]. A decrease in the intracellular pool of guanine nucleotides also reduced the GTP-bound Rheb level, inhibiting mTORC1 activity [23]. Indeed, the therapeutic effect of purine biosynthesis inhibitors may be related to the inhibition of mTORC1 signaling in cancer. Furthermore, experimental evidence suggested that mTORC1 regulates the intracellular nucleotide pool available for the synthesis of nucleic acids by increasing the metabolic flux through de novo purine synthesis in the mitochondria [21]. Interestingly, intracellular bodies that organize purine biosynthetic enzymes, called purinosomes, colocalize with the mitochondria, and their assembly is influenced by mTORC1 [24]. Moreover, activation of the mTORC1 signaling pathway induces the phosphorylation of CAD (carbamoyl phosphate synthetase 2, aspartate transcarbamylase, dihydroorotase), an enzyme important for the early steps of de novo pyrimidine synthesis [25].

**Figure 1:**
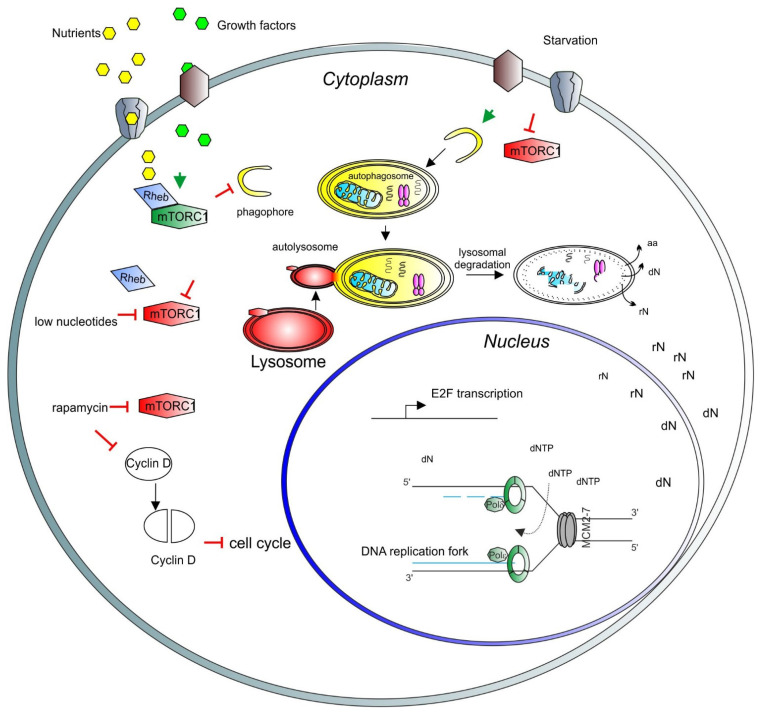
A model depicting the role of mTORC1 in the cell cycle and nuclear activity regulation. Growth factors (light green hexagons) and nutrients (yellow hexagons) enter the cell through the membrane channels or bind and activate membrane receptors (brown and gray shapes), activating the mTORC1 complex (green long hexagon) via Rheb (blue rhomboid). Activated mTORC1 inhibits catabolic pathways, such as autophagy and lysosomal degradation. Upon starvation, mTORC1 is inhibited (red long hexagon) and autophagy can be triggered. Early stages of autophagy involve phagophore formation (yellow half-circle), followed by the formation of the autophagosome containing trapped cargo (yellow oval). The lysosome (red oval) then fuses with the autophagosome to form the autolysosome (red and yellow oval). Finally, the cargo is degraded, and recycled products are released to the cytosol for further use (aa = amino acids, rN = ribonucleosides, dN = deoxyribonucleosides). In the nucleus, nucleosides are converted to ribonucleotide triphosphates and deoxyribonucleotide triphosphates (dNTP) to be used for transcription and DNA synthesis, respectively. In the nucleus, a basic unit of DNA synthesis, called replication fork, is depicted. Newly synthesized DNA is shown as blue lines, the DNA polymerase complex is shown as green octagons and the DNA helicase complex MCM2-7 is shown as a grey barrel. A low level of nucleotides inhibits mTORC1. Rapamycin inhibits also mTORC1 activity, which induces degradation and accumulation of the D-type cyclins, negatively affecting the cell cycle progression. As a result, transcription of E2F and its target genes is inhibited. E2F activity is necessary for the transcription of genes involved in deoxynucleotide metabolism.

The mTOR selective inhibitor, rapamycin, was initially discovered as an antifungal compound [26] and is now widely used as an immunosuppressant after organ transplantation [27]. Rapamycin is also used as mono- and combination therapy for cancer treatment [28]. De novo nucleotide biosynthesis regulated by mTORC1 might affect the immune system activity. Indeed, mTORC1 activation regulates changes in T-cell metabolism that allow differentiation and expansion of these cells [29]. It would be interesting to investigate whether the antitumor and immunomodulatory effects of the mTORC1 inhibitors are related to the synthesis of nucleotides (Figure 1).

**Figure 2:**
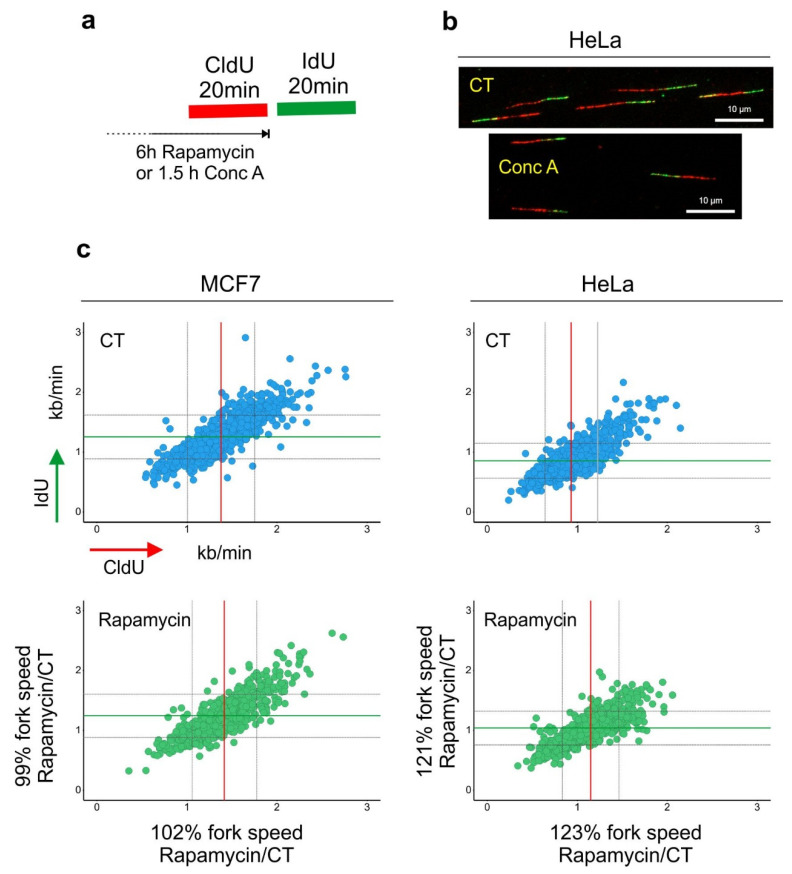
Lysosomal function and DNA replication fork progression. (**a**) An experimental diagram of the DNA replication fork speed analysis. (**b**) Representative images of double-labeled DNA fibers are shown. Cells were pulse-labeled with CldU (red) for 20 min, washed with the medium and pulse-labeled with IdU (green) for a subsequent 20 min. Cells were lysed and their DNA was stretched. Nucleosides were detected with antibodies, and the length of CldU and IdU pulses (µm) was converted into kb/min as in [39]. Before the labeling, cells were incubated for 6 h with rapamycin (100 nM). (**c**) The length of every replication fork was converted into kb/min and is plotted on the graphs. Dataset for MCF7 cells was taken from [30]. Control (CT) mean fork speed: CldU = 1.37 kb/min and IdU = 1.25 kb/min; number of scored forks: *n* = 758. Rapamycin-treated mean fork speed: CldU = 1.41 kb/min and IdU = 1.24 kb/min; number of scored forks: *n* = 557. HeLa cells, control (CT) mean fork speed: CldU = 0.93 kb/min and IdU = 0.85 kb/min; number of scored forks: *n* = 825. Rapamycin-treated mean fork speed: CldU = 1.14 kb/min and IdU = 1.03 kb/min; number of scored forks: *n* = 544. The red line indicates the mean fork speed of the CldU pulse, and the green line indicates the mean fork speed of the IdU pulse. Grey lines indicate SD. Indicated percentages of the fork speed were obtained by calculating the fork speed ratio control/rapamycin. Rapamycin treatment accelerated the speed of fork progression in HeLa cells.

It is likely that the degree to which mTORC1 signaling influences the synthesis of purines and pyrimidines varies between cell types depending on whether the cells predominantly use de novo synthesis or the salvage pathway to replenish their intracellular nucleotide pools. Furthermore, the drop in the nucleotide precursor level reported upon rapamycin treatment appears to be a long-term effect of mTORC1 inhibition [21]. In a recent study, we found that incubation of cells for 6 h in the presence of rapamycin induced accumulation of LC3-positive vesicles but did not negatively affect DNA synthesis. On the contrary, in HeLa cells, rapamycin slightly increased the speed of DNA replication [30]. Presumably, inhibition of mTORC1 triggers catabolic pathways, such as autophagy, to balance the availability of intracellular nucleotides (Figure 2). Moreover, the long-term effect of mTROC1 inhibition on DNA synthesis could be an indirect result of cell cycle arrest.

In eukaryotic cells, the cell cycle is primarily controlled by the activation of cyclin-dependent kinases (Cdks) Cdk4/Cdk6 and Cdk2 by their protein-binding partners, cyclins D1–3, cyclin E and cyclin A. D-type cyclins (D1, D2 and D3) are expressed in a linage-dependent fashion. Upon growth factor stimulation, expression of cyclin D, which is necessary for the G1 progression [31], is enhanced. Overexpression of cyclin D1 has been reported in many different tumors [32]. Cyclin D1 forms a kinase complex with Cdk4 and Cdk6 to allow the cell to progress through the G1 phase by phosphorylating Rb. This phosphorylation of Rb induces its inactivation. The full progression through the S phase requires the phosphorylation of Rb to release the transcription factor E2F [33]. Rapamycin treatment delayed the accumulation of cyclin D1 transcripts and affected their stability. Together, rapamycin decreased the level of the cyclin D1 protein. This effect was more evident after 8 h of rapamycin treatment [34]. Therefore, the long-term effect of mTORC1 inhibition on DNA synthesis might be strengthened by other mechanisms of cell cycle arrest, such as downregulation of cyclin D1 and E2F.

During the S phase, copies of genetic information are produced in the process of DNA replication. This process is regulated with high precision, as any defects might compromise genomic integrity. Proliferating human cells have a diploid genome (2n = 46 chromosomes) with approximately 6 × 10^9^ base pairs of DNA. The duplication process proceeds from origins of replication scattered throughout the genome [35]. Origins of replication are of fundamental importance to ensure that DNA is copied only once during each cell cycle [36]. In humans, origins of replication seem to contain a G-rich DNA sequence signature that can be recognized by the origin recognition complex (ORC) [37]. The ORC complex directs the assembly of other factors, such as Cdc6, Cdt1 and the MCM2-7 complex, to form a prereplication complex (pre-RC). Pre-RC is assembled during the late G1 phase of the cell cycle and serves to direct a single round of DNA synthesis. Once the cell has passed the restriction point in G1 and engaged into the S phase, the Cdks/cyclin complex activates the helicase function of the MCM2-7 complex, and DNA synthesis proceeds with the recruitment of DNA polymerases and other regulatory factors [38]. It has been shown that rapamycin inhibits mRNA and protein expression of MCM6 and 7. Unsurprisingly, the inhibitory effect of rapamycin was reversed by overexpression of E2F. The latter indicates that the effect of mTORC1 inhibitors on DNA synthesis could be, at least partially, a result of general cell cycle arrest. Treatment with mTORC1 inhibitors might lead to general metabolic alterations in the cell that later affect the cell cycle progression and DNA replication. To test the immediate role of mTORC1 activity in genomic DNA synthesis, genetic manipulations of the mTORC1 components are needed.

Altogether, available evidence indicates the complexity of the mechanism regulating the nucleotide pool to support DNA synthesis and that the role of mTORC1 in DNA replication could differ, depending on the cell type and the metabolic status of the cell. It would be interesting to measure the level of deoxynucleosides in normal and cancer cells treated with rapamycin for different time points. Besides the clear evidence of mTORC1′s role in integrating proliferative signals with cellular metabolism, future studies are necessary to better understand the interplay between mTORC1 and the metabolic pathways involved in purine and pyrimidine biosynthesis that might influence DNA replication.

## 3. Autophagy and DNA Synthesis

Depending on the mechanism by which the cytoplasmic content is delivered to the lysosomes for degradation, the autophagy processes are known as microautophagy, chaperone-mediated autophagy and macroautophagy [40]. Autophagy operates at basal levels in normal growing conditions to maintain metabolic homeostasis. This is achieved through the degradation of cytoplasmic components, in particular damaged proteins, membranes and organelles [41]. Lysosomal degradation of autophagy cargos not only removes potential hazards from the cytoplasm but also provides substrates for anabolism, as we described in the previous section. Microautophagy involves a direct uptake of cytoplasmic targets to the lysosome or late endosome through membrane invagination. Chaperone-mediated autophagy (CMA) is relevant for protein degradation. Targeted proteins are delivered to the lumen of the lysosome via transmembrane translocation rather than membrane fusion [42]. The protein chaperone HSPA8/HSC70 binds to KFERQ-containing proteins targeted for degradation. This binding allows the import of the to-be-degraded protein into the lysosome through the lysosome-associated membrane protein 2A (LAMP2A) [43].

Macroautophagy (hereafter referred to as autophagy) is perhaps the most complex type of autophagy. It can be divided into four distinct phases: (i) initiation (pre-autophagosomal structure), (ii) nucleation–elongation–maturation (phagophore to autophagosome), (iii) fusion with the lysosome (autophagolysosome) and (iv) degradation (autolysosome) [44]. To date, around 40 autophagy-related genes (ATG) have been discovered in mammalian cells, 16 of which are considered essential for both selective and bulk autophagy. The remaining genes are involved in one or more of the 15 different types of selective autophagy [41]. Interestingly, 99 Atg genes have been identified in planktonic rotifers [45], likely reflecting specific metabolic requirements of these organisms.

Triggers of autophagy include nutrient starvation, oxidative stress and mTORC1 inhibition. Phagophore initiation begins at ATG9-rich membranes with the recruitment of ATG14 and the ATG1/Unc51 like autophagy activating kinase 1 (ULK1) kinase complex (ULK1, ULK2, ATG13, FAK family kinase-interacting protein of 200 kDa (FIP200) and ATG101) [46]. Next, the class III PI3K complexes (ATG14, vacuolar protein sorting 34 (VPS34), VPS15, beclin1 (BECN1), nuclear receptor binding factor 2 (NRBF2), UV radiation resistance gene (UVRAG) and autophagy and beclin1 regulator 1 (AMBRA1)) are recruited. These complexes positively regulate the ULK1 complex and the recruitment of DFCP1 and WD repeat domain, phosphoinositide interacting 2 (WIPI2). The resulting macromolecular complex acts as a scaffold for the formation of ATG12–ATG5:ATG16L1 complexes, which, together with phosphatidylinositol 3-phosphate (PI(3)P), play a crucial role in the elongation step [47]. The formation of the ATG12–ATG5:ATG16L1 complex is catalyzed by the ubiquitin-like conjugation system, consisting of ATG7 and ATG10. ATG3, ATG7 and the conjugation product of ATG7 then participate in another ubiquitin-like conjugation system, which conjugates phosphatidylethanolamine to ATG8 (microtubule-associated protein 1 light chain 3 beta (LC3B)) and five members of the GABA type A receptor-associated protein (GABARAP).

The loss of *Atg* genes involved in the initial steps of autophagosome formation is lethal in mouse embryos. *Atg5*-null mice failed to develop beyond the eight-cell stage; therefore, autophagic activity is essential for preimplantation of the embryo [48]. Furthermore, *Atg*7-null mice showed impaired constitutive and starvation-induced autophagy, and the animals died shortly after birth [49]. While these results indicate that autophagy is necessary for normal development, ATG proteins can potentially also have roles in pathological conditions. The contribution of autophagy to the pathogenesis of neurodegenerative diseases, inflammatory diseases and aging is well documented [50]; however, the relationship between autophagy and cancer is still a matter of discussion. Autophagy may promote tumor survival and metabolic adaptation by providing nutrients to survive in hypoxic conditions. During starvation, autophagy can reduce ROS levels by removal of damaged mitochondria, thereby counteracting potential DNA damage [51]. Confirming the role of autophagy in promoting cancer survival, the inhibition of autophagic flux induced apoptosis and inhibited cell proliferation in breast and hepatic cancer cell lines [52]. The association between tumor cell survival and autophagy can also be explained by the fact that autophagy may protect cells from apoptosis; however, it can be cell-context-dependent [53]. Such context-dependent effects need to be further characterized, highlighting the importance of a better understanding of the molecular mechanisms that determine how autophagy impacts the cell cycle progression.

On the contrary, a line of evidence suggests that defects in autophagy could lead to tumorigenesis. Indeed, the deletion of *BECLIN* has been observed in human breast, ovarian and prostate cancer [54]. Autophagy is also necessary for the acquisition of the senescence phenotype [55]. Cellular senescence has been defined as a state of stable cell cycle arrest with an active metabolism. Oncogene-induced senescence (OIS) illustrates well the tumor-suppressive role of senescence [56]. Confirming the role of autophagy in cell cycle arrest, incubation with the genotoxic drug, temozolomide, resulted in increased autophagic flux before the acquisition of senescence [57], indicating that autophagy might protect from cancer development. Available results suggest that potential cancer therapy based on autophagy inhibition must take into consideration the underlying contexts, in which such inhibition would be beneficial and not detrimental. Nevertheless, as cancer cells are far more dependent on autophagy for survival than normal cells, there might be a therapeutic window for the use of autophagy inhibitors for cancer treatment [58].

Of relevance to genome integrity maintenance, there is experimental evidence suggesting that autophagy might have a role in DNA repair. Cells with compromised autophagic flux seem to rely more on the error-prone nonhomologous end-joining (NHEJ) repair pathway [59]; however, their response to exogenous DNA damage has not been yet investigated. The role of autophagy in DNA synthesis and repair has been studied so far mainly in yeast. It has been shown that autophagy impacts the nucleotide pools after DNA damage via selective degradation of the ribonucleotide reductase 1 (RNR1). This protein is critical for the reduction of ribonucleotides to deoxyribonucleotides (dNTPs), a crucial step in the DNA synthesis process [60]. It is reasonable to speculate that in mammalian cells, autophagy could help ensure a correct concentration of dNTPs. A lower level of dNTPs or altered relative proportion of the dNTP concentration compromises genome integrity by triggering DNA replication stress [61].

Mitochondria are considered the main source of endogenous reactive oxygen species (ROS), which can damage DNA. If the lesions remain unrepaired, they might compromise mitochondrial DNA and genome integrity. Selective autophagy of mitochondria (mitophagy) plays a key role in the degradation of damaged mitochondria. ROS have been also proposed to act as an important signal that triggers autophagy during starvation. Nutrient deprivation results in increased ROS production in the mitochondria due to metabolic stress. Elevated ROS can cause DNA damage, and in turn, autophagic flux is increased as a part of the DNA damage response (DDR) [62].

**Figure 3:**
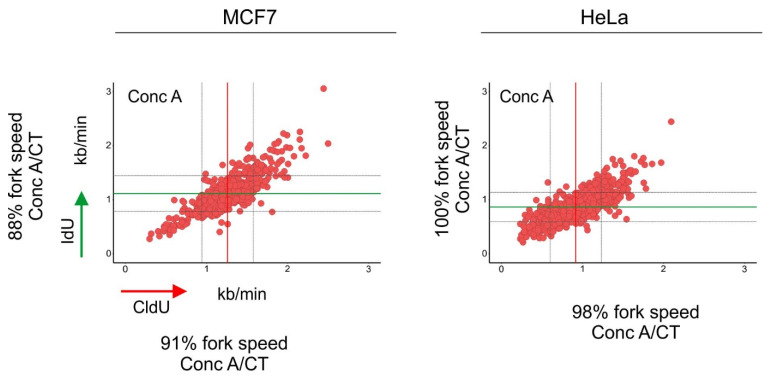
Inhibition of autophagic flux reduced the speed of DNA replication fork progression. Cells were pulse-labeled with CldU (red) for 20 min, washed with the medium and pulse-labeled with IdU (green) for a subsequent 20 min. Cells were lysed and their DNA was stretched. Nucleosides were detected with antibodies, and the length of CldU and IdU pulses (µm) was converted into kb/min as in [39]. Before the labeling, cells were incubated for 1.5 h with concanamycin-A (Conc A; 2 nM). The length of every replication fork was converted into kb/min and is plotted on the graphs. Dataset for MCF7 cells was taken from [30]. Conc A-treated mean fork speed: CldU = 1.25 kb/min and IdU = 1.11 kb/min; number of scored forks: n = 615. HeLa cells, Conc A-treated mean fork speed: CldU = 0.91 kb/min and IdU = 0.86 kb/min; number of scored forks: *n* = 751. The red line indicates the mean fork speed of the CldU pulse, and the green line indicates the mean fork speed of the IdU pulse. Grey lines indicate SD. Indicated percentages of the fork speed were obtained by calculating the fork speed ratio control/concanamycin-A. Control values of the fork speed from untreated cells are shown in Figure 2. Concanamycin-A treatment reduced the speed of fork progression in MCF7 cells.

Recently, we systematically evaluated the relationship between autophagy and DDR by (i) inducing DNA damage with drugs that cause single- and double-strand DNA breaks, (ii) evaluating the extent of DNA damage and repair in cells with defective autophagy and (iii) measuring the dynamics of autophagy upon DNA damage. We consistently showed that after DNA damage, DDR was activated first, and shortly after, we were able to detect autophagy induction. Preaccumulation of autophagy markers by treating cells with rapamycin, followed by genotoxic insults, seemed to accelerate DNA repair. Interestingly, the knock-out of either *ATG5* or *ATG7* delayed DNA repair in MCF7 cells, but no differences were observed in HeLa cells. These results confirm that autophagy is necessary for the repair of DNA; however, it might occur in a cell-context-dependent manner. Moreover, we observed that both knock-out cell lines lacking *ATG5* or *ATG7* experienced DNA replication stress, which was alleviated by supplementation with exogenous deoxynucleosides. It is worth noting that the inhibition of autophagic flux using concanamycin-A impacted negatively genomic DNA synthesis in both MCF7 and HeLa cells [30] (Figure 3). Autophagy may play an important role in normal DNA synthesis, especially in cells with limited nucleotide pools; however, further research is warranted to confirm this hypothesis. One of the important questions lacking experimental evidence is whether any of the subunits of the mammalian ribonucleotide reductase (RNR1 and 2) are regulated directly by autophagy.

## 4. Recycling of Nucleic Acids

Being a treasury containing the genetic material, the nucleus of eukaryotic cells has been seen as the master regulator of cellular functions. A growing body of evidence suggests that there is a complex crosstalk between many cell organelles and the nucleus to control metabolic homeostasis and cell survival [63]. Several metabolic stressors can compromise nuclear functions and, ultimately, genomic integrity. Like any other component in the cell, nuclear content can be targeted by autophagy. One of the first observations of the role of autophagy in targeting nuclear structures came from experiments performed in yeast. Vacuoles (the equivalent of mammalian lysosomes in yeast) that merged with the nuclear membrane seen in yeast presumably promoted the degradation of the nuclear envelope and likely other nuclear components [64]. In mammalian cells, autophagy degrades the nuclear envelope protein lamin B1 (LMNB1), which is mediated by the direct interaction between LC3B and LMNB1. This interaction might protect cells from tumorigenesis by degrading the nuclear envelope in response to DNA damage [65]. This interesting observation opens the possibility that autophagy could safeguard genomic integrity by targeting different nuclear structures for degradation, as discussed below.

Defects in autophagy could lead to the accumulation of DNA damage [30,54]. Whether autophagy targets the chromatin directly or indirectly is a matter of current research. To date, the role of autophagy in micronuclei degradation is well-documented [66]. Micronuclei formation can be a consequence of errors during DNA synthesis, such as incomplete DNA replication during the S phase, and/or problems in chromosome segregation during mitosis. Both of these phenomena can be caused by replication stress [67,68]. Interestingly, it has been reported that a significant percentage of micronuclei in cancer cells contain LC3 puncta, and this LC3/micronuclei colocalization is lost after knock-down of either *ATG5* or *ATG7*. This indicates that micronuclei (or at least a proportion of them) are degraded by autophagy [69]. The size and the number of micronuclei that can be targeted by autophagy remain to be determined. Another open question is whether autophagy-mediated micronuclei degradation contributes to the re-establishment of genomic integrity. Furthermore, whether the recycling of the chromatin contained in micronuclei contributes to anabolic processes in the cell, such as nucleotide and amino acid biosynthesis, is still unknown.

Active transcription and protein synthesis are necessary for cell survival and growth. More than half of intracellular nucleotides are contained in the ribosomes in the form of rRNA molecules. There is evidence suggesting that mTORC1 is a regulator of ribosome biogenesis [70]. An increase in cell growth is accompanied by higher demand for ribosome biogenesis and elevated usage of nucleotides, which must be acquired by de novo synthesis or exogenous uptake; therefore, the recycling of intracellular RNA might be important to maintain normal cellular homeostasis but could also be a target in highly proliferative cancer cells.

There are direct mechanisms that involve lysosomes in nucleotide degradation. The lysosomal lumen contains several enzymes, among them RNase T2 and DNase2a. RNase T2 cleaves single-stranded RNA into mono- and oligonucleotides [71], whereas DNase2a is an endonuclease that cuts double-stranded DNA with low or null sequence specificity [72]. RNautophagy targets RNA for its direct transport through the lysosomal membrane, leading to lysosomal degradation. This pathway involves SIDT2 and the lysosomal membrane protein LAMP2C [73]. In yeast, starvation induced RNA degradation, and the resulting nucleosides were further converted into nucleotides [74]. Nevertheless, the effect of RNautophagy inhibition on the homeostasis of intracellular nucleotide pools in human cells remains to be investigated.

DNautophagy has been defined as the type of autophagy specialized in the degradation of DNA. In this process, DNA is directly taken up by lysosomes and degraded. In addition to its role in RNautophagy, LAMP2C can interact directly with DNA [75]. Furthermore, deficiencies in lysosomal DNA-degrading enzymes lead to the accumulation of self-DNA and induction of autoimmunity in mice. Indeed, mice deficient in *Dnase2a* showed impaired removal of cytoplasmic damaged DNA, similarly to autophagy-deficient cells [76]. This accumulation of cytosolic DNA triggered inflammation via the *Sting* pathway [77].

It has been proposed that DNautophagy and RNautophagy are mechanisms that protect cells from exogenous nucleic acids, most commonly from viral infections. After contacting the host cell, almost all enveloped viruses enter the cytoplasm via endocytosis. To establish a productive infection, viruses have to deliver their genetic material to the host’s cytoplasm and nucleus, while escaping from degradation and overcoming the protective cellular mechanisms. Once viruses are taken into the cell, they are transported via membrane trafficking to the lysosomes. In the lysosomes, viruses undergo uncoating, and their genetic material is released into the cytoplasm and transported into the nucleus [78]. It is not surprising that viruses have developed specific strategies to avoid nucleotide degradation. A recent example is SARS-CoV-2, which evades lysosomal destruction by a mechanism dependent on the viral ORF3a inhibiting autophagy. ORF3a interacts with the autophagosomal protein STX17. This interaction blocks the assembly of the STX17–SNAP29–VAMP8 SNARE complex, inhibiting the fusion of autophagosomes with lysosomes [79].

**Figure 4:**
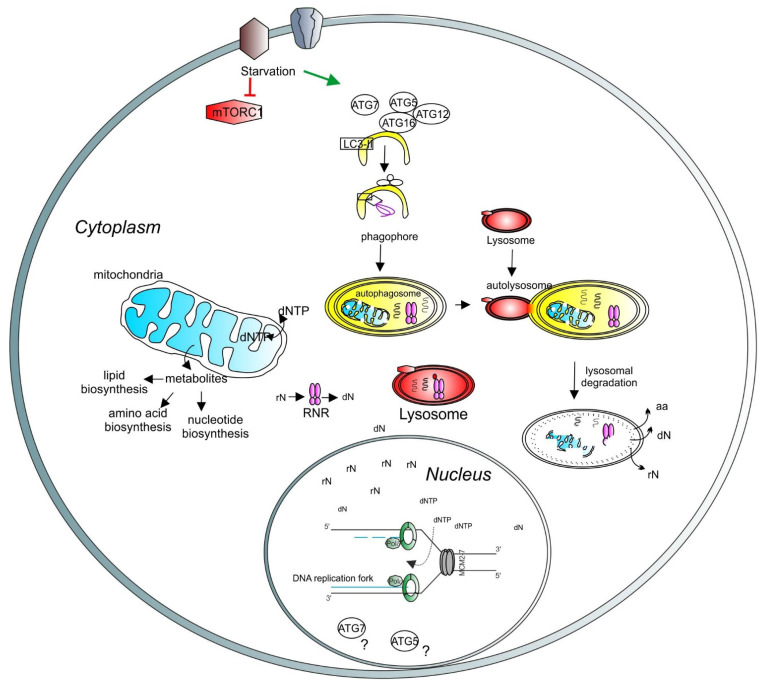
A model depicting the role of lysosomes and autophagy in DNA synthesis and transcription. Lysosomal function is regulated by mTORC1 (red hexagons). Under starvation, autophagy is initiated by ATG proteins (empty circles) and LC3-related processes (empty square) to form the phagophore (yellow half-oval). The cargos are engulfed in the autophagosome, which then fuses with the lysosome to create the autolysosome, where the cargos are degraded. Recycled molecules are released to the cytoplasm for further use. Defective cellular organelles, such as mitochondria (blue shape), can be also targeted for degradation by autophagy. Healthy mitochondria contribute to the correct balance of intracellular metabolites. Intracellular nucleotide pools (ribonucleotides (rNTP) and deoxyribonucleotides (dNTP)) are important for maintaining adequate levels of DNA synthesis and transcription. Lysosomes can directly target the ribonucleotide reductase (RNR, pink shape) for degradation, thus regulating the deoxynucleotide level. A basic unit of DNA synthesis, called replication fork, is shown with DNA polymerases (green shapes) and the helicase MCM2–7 (grey shapes). The role of autophagy-related proteins, such as ATG5 and ATG7, in the regulation of the nuclear function requires further investigation (question marks).

Some of the anabolic processes involved in nucleotide production require healthy mitochondria [80]. The electron transport chain and ATP production (OXPHOS) generate ROS in the mitochondria. An excessive amount of ROS could negatively affect mitochondrial membranes, lipids and DNA (mtDNA). Thus, the removal of damaged mitochondria might be important to maintain metabolic homeostasis. Mitophagy receptors or ubiquitin chains attached to specific proteins on the mitochondrial surface are exposed upon mitochondrial stress and tag mitochondria for degradation. These degradation tags recruit the autophagy machinery to the mitochondrial surface. An autophagosome is formed to engulf tagged mitochondria and merges with the lysosome. Mitochondria are then degraded by the enzymes contained in the lysosome. The maintenance of a healthy mitochondria pool can contribute to the correct balance of the intracellular nucleotide level (Figure 4).

## 5. Perspectives

Lysosomes are at the crossroad of degradative pathways, endocytosis and autophagy. Being involved in the exclusion and degradation of infectious agents and self-DNA, lysosomes are important to trigger innate and adaptive immunity [81]. Impairment of lysosomes may affect the mitochondrial function and lead to the accumulation of damaged mitochondria, as often observed in lysosomal storage disorders (LSDs). To date, approximately 50 LSDs have been described, and most of the related mutations have been elucidated [82]. One of the LSDs, Niemann–Pick disease, is caused by the loss of function of distinct lysosome-residing proteins, the acid sphingomyelinase and NPC1. The stability of the lysosomes is compromised as a consequence of mutations in SMPD1, which encodes the acid sphingomyelinase [83]. One of the defects seen in this disease is the accumulation of cholesterol in mitochondria, resulting in mitochondrial dysfunction and defective antioxidant defense [84]. By increasing the stability of the lysosomes, some of the defects caused by Niemann–Pick disease can be corrected [85]. Interestingly, the DNA damage response seems to be altered in cells from Niemann–Pick disease patients, and these cells are more resistant to apoptosis after gamma radiation [86]. It is currently unknown whether LSDs can cause direct or indirect problems in DNA synthesis; however, it is reasonable to speculate that some of the LSDs might lead to replication stress because of metabolic imbalance, ultimately compromising genome integrity [87].

The cationic molecule, acridine orange (AOH+), is a nucleic acid binding dye that emits green fluorescence when bound to double-stranded DNA and red fluorescence when bound to single-stranded DNA or RNA. AOH+ induces the condensation of nucleic acids in solutions. As a DNA-intercalating molecule, it might have mutagenic effects [88]. AOH+ has been also used to stain lysosomes, giving a strong orange signal under the fluorescence microscope. The accumulation of AOH+ in different cellular structures seems to be pH-dependent [89]. There is a possibility that cationic drugs targeting the lysosome [90] could affect nuclear functions, an intriguing hypothesis worth further exploration.

Published evidence suggests that lysosomal function is critical for metabolic homeostasis maintenance. Lysosomes can integrate anabolic and catabolic pathways to regulate cell growth and proliferation. Together with autophagy, lysosomes can regulate the availability of nutrients for basic cellular functions, such as nucleotide biosynthesis and energy homeostasis, both necessary for correct DNA synthesis and transcription. Therefore, targeting lysosome function opens new avenues to improve health and fight against diseases.

## Data Availability

The data presented in this study are available on request from the corresponding author.
